# Synaptonemal Complex Length Variation in Wild-Type Male Mice

**DOI:** 10.3390/genes1030505

**Published:** 2010-12-15

**Authors:** Neil M. Vranis, Godfried W. van der Heijden, Safia Malki, Alex Bortvin

**Affiliations:** 1 Johns Hopkins University, Baltimore, MD 21218, USA; E-Mail: nvranis1@jhu.edu; 2 Department of Embryology, Carnegie Institution for Science, Baltimore, MD 21218, USA; E-Mails: gwvanderheijden@gmail.com (G.W.H.); malki@ciwemb.edu (S.M.)

**Keywords:** synaptonemal complex, meiosis, mouse, genetic background, synapsis, REC8

## Abstract

Meiosis yields haploid gametes following two successive divisions of a germ cell in the absence of intervening DNA replication. Balanced segregation of homologous chromosomes in Meiosis I is aided by a proteinaceous structure, the synaptonemal complex (SC). The objective of this study was to determine total average autosomal SC lengths in spermatocytes in three commonly used mouse strains (129S4/SvJae, C57BL/6J, and BALB/c). Our experiments revealed that the total autosomal SC length in BALB/c spermatocytes is 9% shorter than in the two other strains. Shorter SCs are also observed in spermatocytes of (BALB/c × 129S4/SvJae) and (C57BL/6J × BALB/c) F1 hybrids suggesting a genetic basis of SC length regulation. Along these lines, we studied expression of a selected group of genes implicated in meiotic chromosome architecture. We found that BALB/c testes express up to 6-fold less of *Rec8* mRNA and 4-fold less of REC8 protein. These results suggest that the mechanism that defines the SC length operates via a REC8‑dependent process. Finally, our results demonstrate that genetic background can have an effect on meiotic studies in mice.

## 1. Introduction

Generation of haploid gametes from diploid germ cells is accomplished by way of meiosis, a specialized process comprised of two cell divisions lacking intervening replication of the genome. Precise segregation of homologous chromosomes poses a major logistical challenge for the germ cell. This difficult problem is elegantly solved by bringing homologs in physical proximity and cementing their associations by means of the synaptonemal complex (SC) [1,2,3,4,5]. This structure facilitates the formation of crossovers (COs) of which chiasmata are the end product [6]. After finalization of COs, the SC dissolves; the resulting bivalents are oriented on the metaphase plate and subsequently pulled apart into daughter cells.

In this report, we focused on mouse spermatocytes and, specifically, on the physical length of the SC, a proteinaceous structure prominently featuring between homologous chromosomes in pachynema of meiotic prophase I. It has been shown previously in several species that the length of the SC positively correlates with the number of COs [7,8]. This is likely the consequence of a mechanism named CO interference [9]. As a consequence, COs are more evenly spaced throughout the genome [10,11].

The SC consists of two lateral elements (LEs), a central region or element (CE) and transverse filaments (TFs) [1,2,3,4,5]. Lateral elements of the SC are structural descendants of axial elements (AE) (chromosomal axes) that develop during leptonema and are comprised of cohesin complexes, coiled‑coil domain proteins, and the bases of large loops of genomic DNA. As diploid germ cells cease proliferating and initiate meiosis, most mitotic cohesin complex proteins are superseded by meiotic counterparts [12]. For example, mitotic cohesin subunit SMC3 remains active in meiosis but the function of SMC1α protein is largely carried out by SMC1β [13,14,15]. Likewise, mitotic cohesin subunits SCC1 and SCC3 are replaced by REC8 and STAG3, respectively [16,17]. Two coiled-coil domain proteins, SYCP2 and SYCP3, are exclusive to meiotic axes and are essential for AE/LE and SC functioning in the mouse male germline [18,19,20]. In zygonema, AEs of homologous chromosomes gradually align and develop discernible SCs that contain SYCP1, a TF component, and later incorporate SYCE1, SYCE2, and TEX12 proteins that function in the CE [1,21,22,23,24,25]. 

AE/LEs serve as attachment sites for bases of loops of chromosomal DNA but the mechanism of such interaction remains to be elucidated [26,27,28,29]. One possibility is that AE/LE proteins interact directly with DNA either in a sequence-specific or non-sequence-specific manner. Cohesin complexes might be early and even sole determinants of such interactions. These early interactions might be further strengthened or amended by SYCP2 and SYCP3 proteins that have putative DNA-binding properties. The few attempts to identify DNA sequences associated with chromosomal axes in rodents did not reveal any common sequence motifs but suggested enrichment of repeated, transposon derived DNA (LINE, SINE) [28,30,31,32]. Alternatively, AE/LE interaction with genomic DNA might be indirect and mediated by either constitutive or meiosis-specific chromatin-associated proteins. 

Regardless of the mechanism of AE/LE association with DNA, the degree of chromosome compaction ultimately should be reflected in the length of the SC. Thus, careful comparison of SC lengths between various genetic mutants and even among wild-type organisms might provide clues to both structural and functional aspects of SC and meiotic chromosomes [33,34]. In this study, we compared lengths of SCs in spermatocytes of different wild-type mouse strains. By studying SCs in spermatocytes of three widely used laboratory mouse strains (129S4/SvJae, C57BL/6J and BALB/c), we determined that the total average autosomal SC length in one particular strain, BALB/c, is approximately 9% shorter that those of 129S4/SvJae and C57BL/6J mice. Furthermore, by studying SC lengths in spermatocytes of (BALB/c × 129S4/SvJae) F1 and (C57BL/6J × BALB/c) F1 animals, we obtained evidence that suggested a genetic basis for the short SC length. Finally, results of expression analysis of a subset of SC-linked genes suggested that variation in SC length between strains might be due to lower expression levels of meiotic cohesin subunit REC8 in BALB/c mice. 

## 2. Results

### 2.1. Short SCs in BALB/c Spermatocytes

To examine the possibility of natural variation of SC length among wild-type mice of three pure genetic backgrounds, we prepared nuclear surface spreads of testicular cells and imaged SCs of pachytene spermatocytes by immunofluorescent labeling and microscopy. Meiotic spreads were prepared from pooled testicular cell suspensions of two age-matched males of each of 129S4/SvJae, C57BL/6J, and BALB/c strains, and probed with antibodies to SYCP2, an AE/LEs component [19]. Pachytene nuclei were further studied in detail using MetaMorph to determine the total length of meiotic chromosome axes.

Given the critical importance of the spreading technique for our study, we first wanted to determine if the process of spreading of meiotic chromosomes on glass slides could bias SC measurements and skew the outcome of the experiment. To address this point, we measured surface area occupied by DAPI-stained genomic DNA of individual nuclei and asked if these values positively correlated with measurements of the total SC length. Such analysis was performed for all three strains of interest. The results of such measurements established that the spreading technique had no appreciable positive correlation with the total SC length [correlation coefficients were 0.018 for 129S4/SvJae (*n* = 14), −0.131 for C57BL/6J (*n* = 20) and 0.364 for BALB/c (*n* = 18) mice]. Thus, the principle method of preparation of meiotic spreads for subsequent analyses should not influence the precision of our observations. 

**Figure 1:**
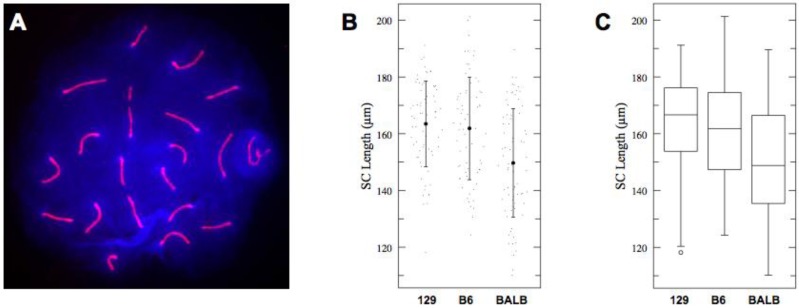
(**A**) Immunofluorescent detection of meiotic chromosome cores (synaptonemal complexes) with anti-SYCP2 antibody (red). Sex chromosomes occupy a separate nuclear territory (the sex body; since synapsis is limited only to the PAR and occurs at late pachytene). The remaining 19 autosomal bivalents exhibit complete synapsis. Genomic DNA was detected with DAPI (blue); (**B**) Total length of autosomal SCs of 129S4/SvJae, C57BL/6J and BALB/c spermatocytes. Shown are actual values (dots), mean values (solid circles) of SC lengths with one standard deviation (whiskers); (**C**) Box-plot representation of SCs lengths in populations of 129S4/SvJae, C57BL/6J and BALB/c spermatocytes. Shown are median, interquartile range (IQR), whiskers correspond to maximal and minimal data. When suspected outliers are present (unfilled circle in 129S4/SvJae sample) whiskers correspond to 1.5 IQR.

For our measurements, we decided to exclude sex chromosome SCs from our analysis and focus on the total autosomal SC length per individual nuclei. When compared to the autosomes, the SC of the heteromorphous sex chromosomes is known to be more dynamic [35]. The results of measurements and statistical analysis of average total autosomal SC lengths in spermatocytes of each genetic background are summarized in Figure 1 and Table 1. Spermatocytes of 129S4/SvJae and C57BL/6J animals showed no statistically significant difference in the total average length of their autosomal SCs. In contrast, the total average length of meiotic axes in BALB/c spermatocytes was found to be 7.9% and 9% shorter than those of C57BL/6J and 129S4/SvJae, respectively (one-way ANOVA *p* < 0.0001, *F* = 13.36). Thus, despite a certain degree of variability of the SC length within each examined genetic background (as evident from the data in Figure 1), the total autosomal SC length in BALB/c animals is distinctly reduced compared to the other two strains. 

**Table 1:** Total autosomal SC lengths (ANOVA: *p* < 0.0001, *F* = 13.36).

Strain	*n*	Mean	SD	95% CI
129S4	69	163.48	15.1	159.3–167.7
C57BL6J	68	161.89	18.1	157.7–166.1
BALBc	75	149.69	19.2	145.7–153.7

**Figure 2:**
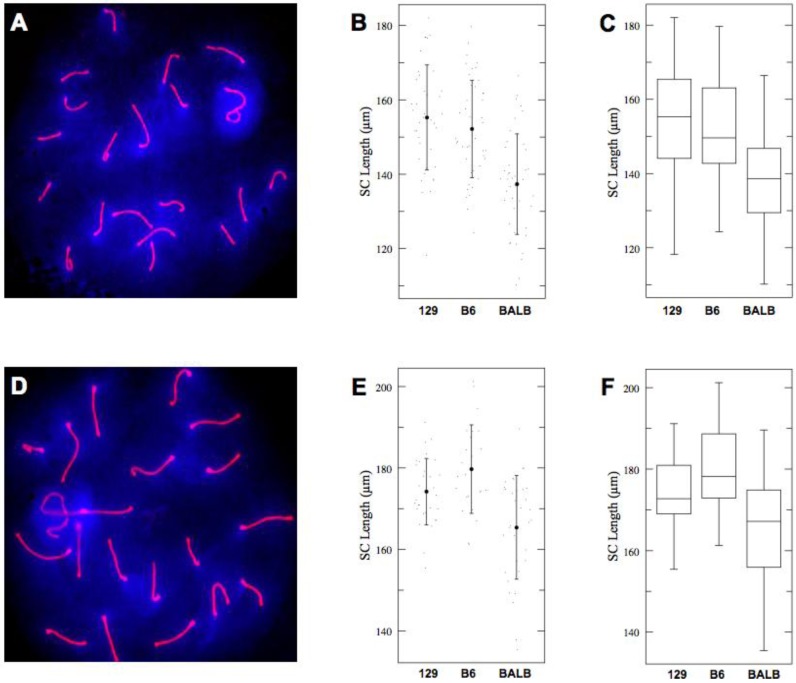
(**A**), (**D**) Examples of “Early” (A) and “Late” (D) pachytene spermatocytes. Red–SYCP2, blue–DAPI; (**B**), (**E**) Total lengths of autosomal SCs in “Early” (B) and “Late” (E) pachytene spermatocytes of three genetics backgrounds. Shown are actual values (dots), mean values (solid circles) of SC lengths with one standard deviation (whiskers); (**C**), (**F**) Box-plot representation of cumulative length of autosomal SCs in “Early” (C) and “Late” (F) pachytene spermatocytes. Shown are median, IQR, maximal and minimal data points.

SCs change their appearance (thickness and length) in the course of pachynema. Therefore, the entire population of pachytene spermatocytes can be subdivided into “early” and “late” groups (see Materials and Methods for details). This circumstance provided us with an opportunity to determine whether shorter SCs of BALB/c spermatocytes predate or, instead, emerge during pachynema. Examination of SC lengths in the two subgroups of spermatocytes revealed that both “early” and “late” pachytene spermatocytes in BALB/c mice had shorter SCs compared to 129S4/SvJae and C57BL/6J males (Figure 2 and Table 2). Thus, the difference in SC lengths between the three strains is determined prior to pachynema of meiotic prophase I.

**Table 2:** Total autosomal SCs lengths in “early” (ANOVA: *p* < 0.0001, *F* = 20.20) and “late” (ANOVA: *p* < 0.0001, *F* = 12.79) pachytene spermatocytes.

Strain	*n*	Mean	SD	95% CI
129S4 (early)	39	155.25	14.1	151.0–159.5
C57BL6J (early)	44	152.17	13.1	148.1–156.2
BALBc (early)	42	137.53	13.4	133.4–141.7
129S4 (late)	30	174.18	8.15	170.3–178.1
C57BL6J (late)	24	179.71	10.9	175.3–184.1
BALBc (late)	33	165.41	12.7	161.7–169.2

### 2.2. No Increase in COs despite Longer SCs in C57BL/6J Mice

The SC functions not only as a physical matrix joining homologous chromosomes but also as an environment for meiotic DNA recombination. Having demonstrated that BALB/c pachytene spermatocytes possess shorter SCs compared to 129S4/SvJae and C57BL/6J mice, we asked if shorter SCs have an effect on DNA recombination. Along these lines, we aimed to determine the average number of recombination nodules (by staining for Replication Protein A (RPA) foci) and the number of COs (chiasmata) in the three strains. 

**Figure 3:**
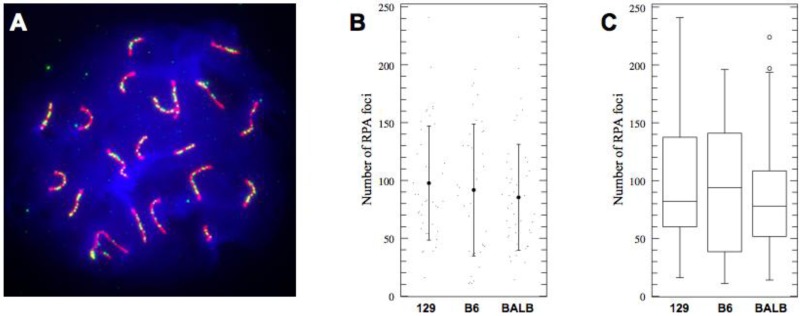
(**A**) Example of RPA localization in “early” pachytene spermatocyte; (**B**) The number of RPA foci does not vary significantly between three pure strains (actual values, mean, and 1 SD shown); (**C**) Box-plot representation of RPA foci distribution in three inbred strains. Shown are median, interquartile range (IQR), whiskers correspond to maximal and minimal data.

RPA is a single-strand DNA-binding protein complex required for the processing of meiotic DSBs [36]. RPA foci are found on meiotic cores in gradually decreasing numbers in the first half of pachynema and thus can be used as DNA recombination marker in pachytene spermatocytes [37,38]. Counting of RPA foci in pachytene spermatocytes of three genetic backgrounds did not provide evidence of statistically significant differences between the examined strains (Figure 3 and Table 3). Thus, the reduced SC length in BALB/c spermatocytes does not result in readily observable reduction of DSB formation frequency. 

**Table 3:** Number of RPA foci in pachytene spermatocytes (ANOVA: *p* = 0.53, *F* = 0.6432).

Strain	*n*	Mean	SD	95% CI
129S4	41	97.68	49.3	82.06–113.3
C57BL6J	37	91.73	57.0	75.29–108.2
BALBc	46	85.39	45.9	70.65–100.1

**Figure 4:**
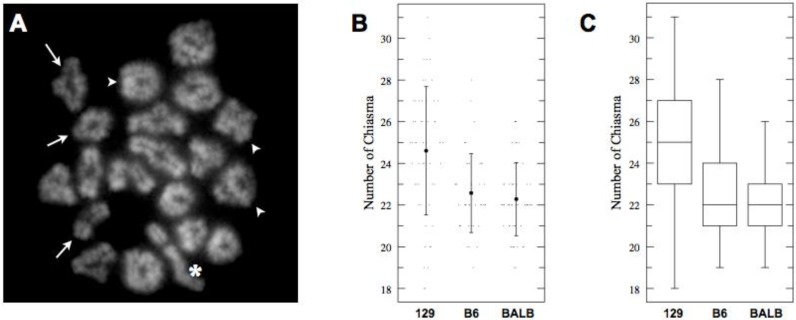
(**A**) Example of Metaphase I spermatocyte (129S4/SvJae). Examples of single and double crossovers are indicated with arrows and arrowheads, respectively. Sex chromosomes are marked with an asterisk; (**B**) Actual values (dots) and mean number of chiasmata observed in Metaphase I spermatocytes in three strains. In contrast to the results of total autosomal SC length measurements, C57BL/6J and BALB/c spermatocytes possess close numbers of crossovers; (**C**) Box-plot of observed chiasma numbers in three pure strains. Shown are median, interquartile range (IQR), whiskers correspond to maximal and minimal data.

Only around 10% of the DSBs generated in meiotic prophase I are converted into COs. These are revealed cytologically as chiasmata in meiotic metaphase I meiocytes. Prior studies in plants, fungi, and metazoans revealed that the SC length of a chromosome positively correlates with its CO numbers [33]. To test whether the difference we observed in SC length between the strains corresponds with a difference in CO numbers, we counted chiasmata joining meiotic chromosomes in metaphase I (Figure 4 and Table 4). This analysis revealed that, consistent with the shorter SCs, the average number of observable chiasmata was statistically lower in BALB/c spermatocytes compared to those of 129S4/SvJae animals. This observation fits well with the notion of coordinated variation in SC length and CO frequency. Intriguingly, however, we have observed that the average chiasmata number in C57BL/6J spermatocytes was close to BALB/c mice despite the significant difference in the SC length between the two strains. This unexpected finding could be explained in terms of CO interference, an as yet molecularly poorly understood process that is responsible for the distribution and frequency of COs along meiotic chromosomes [9,39].

**Table 4:** Chiasmata numbers in three mouse strains (ANOVA: *p* < 0.0001, *F* = 15.19).

Strain	*n*	Mean	SD	95% CI
129S4	53	24.60	3.08	23.97–25.24
C57BL6J	40	22.56	1.89	21.84–23.31
BALBc	56	22.29	1.76	21.67–22.90

### 2.3. The Length of Meiotic Axes Is Potentially Determined Genetically

**Figure 5:**
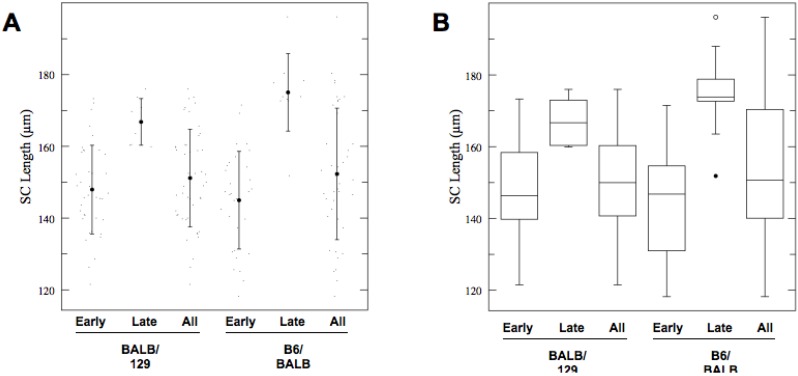
(**A**) Average length of total autosomal SCs in “Early” and “Late” spermatocytes from (BALB/c × 129S4/SvJae) F1 and (C57BL/6J × BALB/c) F1 animals. Shown are actual values (dots), mean values (solid circles) of SC lengths with one standard deviation (whiskers); (**B**) Box-plot representation of the data. Shown are median, interquartile range (IQR), whiskers correspond to maximal and minimal data. When suspected outliers are present (unfilled circle in 129S4/SvJae sample) whiskers correspond to 1.5 IQR.

What is the mechanistic basis for the SC length difference between mouse strains of different genetic backgrounds? To begin to address this question, we determined length of meiotic cores in spermatocytes of (BALB/c × 129S4/SvJae) F1 and (C57BL/6J × BALB/c) F1 animals. These measurements revealed that length of SCs in both types of F1 spermatocytes closely matched that of pure BALB/c males (Figure 5 and Table 5). In addition, as in pure genetic backgrounds, the difference in the SC length between ‘early’ and ‘late’ F1 spermatocytes clearly predated early pachynema. These results imply that both 129S4/SvJae and C57BL/6J chromosomes assume BALB/c-like meiotic configuration and are most consistent with the notion of existence of a dominant allele for short SCs in the BALB/c genome. In the context of this hypothesis, based on sex chromosome composition of F1 males, we exclude a significant role for the Y chromosome in defining short chromosomal axes. 

**Table 5:** Total autosomal SC lengths in F1 hybrid mice (ANOVA: *p* < 0.0001, *F* = 9.101).

Strain	*n*	Mean	SD	95% CI
BALBc/129S4 (early)	39	147.95	12.4	143.4–152.5
BALBc/129S4 (late)	8	166.82	6.51	156.9–176.8
BALBc/129S4(all)	47	151.16	13.6	147–155.3
C57BL6J/BALBc (early)	31	144.98	13.6	139.9–150.0
C57BL6J/BALBc (late)	10	175.05	10.8	166.1–184.0
C57BL6J/BALBc (all)	41	152.31	18.3	147.9–156.7

### 2.4. *Rec8* Expression Is Reduced in BALB/c Mice

**Figure 6:**
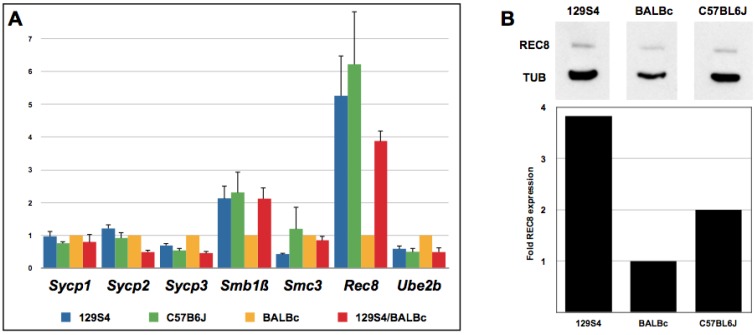
(**A**) Quantitative RT-PCR analysis of a selected group of genes implicated in meiotic chromosome regulation. Shown are relative expression levels using β-actin for normalization; (**B**) Western blot analysis of REC8 expression levels in testes of three pure mouse strains. The blot was probed with antibodies to REC8 and α-Tubulin. Graph shows results of quantification of REC8 levels normalized to α-Tubulin.

Previous analysis of the SCs in mutant meiocytes revealed shorter SCs in *Smc1β* and *Rec8* mutants while SCs in *Sycp3*, and *Ube2b* mutants were longer [10,15,40,41,42]*.* Since our data suggested the genetic basis of short SCs in spermatocytes of BALB/c animals, we examined expression levels in these genes, as well as several others, which have been implicated in meiotic chromosome architecture. We performed quantitative RT-PCR (qPCR) analysis on total RNA samples isolated from testes of adult males of three pure inbred and one mixed F1 genetic backgrounds using primers specific for *Smc1β*, *Rec8*, *Smc3*, *Ube2b*, *Sycp2*, *Sycp3*. Expression of these genes was related to expression of *Act1b*. The results of this analysis (Figure 6A) revealed that one of six tested genes, *Rec8*, is more abundantly expressed in testes of 129S4/SvJae and C57BL/6J than BALB/c animals. Consistent with these results, (129S4/SvJae x BALB/c) F1 animals exhibited intermediate level of *Rec8* mRNA. Taken together, these results suggest that short SCs result from reduced amounts of functional meiotic cohesin complexes in spermatocytes of BALB/c animals. 

To extend this observation to the protein level, we performed Western blot analysis of REC8 and α‑Tubulin expression in testicular protein lysates of three inbred strains (Figure 6B). The results of protein level determination by Western blot revealed ~4- and 2-fold reduction in REC8 levels in BALB/c animals compared with 129S4/SvJae and C57BL/6J, respectively. In general, these results agree with qPCR data and support the notion of a reduced expression of a specialized meiotic cohesin subunit REC8 in BALB/c mice. 

## 3. Discussion

In this work, we determined total average autosomal SC lengths in pachytene spermatocytes of three widely used wild-type strains of mice 129S4/SvJae, C57BL/6J, and BALB/c. Computer-assisted analysis of SCs detected with an antibody to SYCP2 protein revealed that SCs of BALB/c animals are up to 9% shorter than in the other two strains. Furthermore, the difference in the SC length among the three strains precedes early pachynema as both early and late pachytene spermatocytes exhibited similar differences in the SC length. Interestingly, however, while similar in their length, 129S4/SvJae and C57BL/6J chromosome bivalents exhibited contrasting number of COs with C57BL/6J grouping with BALB/c rather than 129S4/SvJae mice. Although this observation should be investigated further, it indicates that C57BL/6J mice possess a more stringent mechanism of CO interference. Our results suggest that the reduction in the length of SCs has an underlying genetic basis since spermatocytes of (129S4/SvJae x BALB/c)F1 and (BALB/c x C57BL/6J)F1 hybrids possessed short SCs reminiscent of those found in pure BALB/c mice. Expression analysis of a selected group of candidate genes revealed differences in mRNA levels for up to 2-fold between the three strains for *Sycp3*, *Smc1β*, *Smc3* and *Ube2b*. The potential effects of these differences on the SC remain unclear. The difference in expression level of REC8 was significantly larger (5–6 times lower in BALB/c when compared to the two other strains). This was reflected in REC8 protein levels. This suggests that shorter SC length in BALB/c animals stems from reduced levels of meiotic cohesin complexes.

Observations made in the course of this study offer insights into several aspects of meiotic chromosomes function in mice. First, our analysis of SC lengths in F1 animals demonstrated that chromosomes of 129S4/SvJae and C57BL/6J backgrounds are readily “reprogrammed” to accommodate shorter SCs. If general notions on meiotic chromosome compaction (that the length of the chromosome axes is defined by the number, size and spacing of chromatin loops) are correct, then our observations suggest that precise sequence identity of genomic DNA associated with AE/LEs are either of no relevance or that such DNA sequences are abundantly present throughout the genome and can be readily adjusted to accommodate the genetic landscape of the host meiotic cell. The latter, for example, will be consistent with prior reports of transposable elements enrichment in SCs. Alternatively, as previously mentioned, this association is not driven by DNA but by chromatin-associated proteins.

Second, our results implicate meiotic cohesin complex and specifically its REC8 subunit in the process that defines the length of the SC. Our finding, that lower expression levels of REC8 are found in a mouse strain that had shorter SCs, is in agreement with the phenotypes of *Rec8* mutant mice. These meiocytes have short AEs that fail to synapse properly and instead accumulate SYCP1 between sister chromatids of individual chromosomes [40,41]. In addition, REC8 orthologs have been implicated in AE assembly and function in various species [43,44,45]. Our findings complement these previous studies of *Rec8* mutants and suggest that SC length is highly sensitive to the levels of meiotic cohesins. Interestingly, REC8 protein is the first cohesin protein detected at the onset of meiosis and shows an axial element-like staining in rat [16]. Potentially DNA loops are already generated by this protein prior to arrival of other axial element components. In light of known covariation between SC length and CO frequency, our results indicate that the latter parameter could also be susceptible to genetic variations harbored by what is considered to be a wild-type genome. At the same time, the observation of unexpectedly lower chiasmata numbers in C57BL/6J spermatocytes could suggest a tighter CO interference in C57BL/6J mice by a mechanism that operates independently from the SC length control in BALB/c mice. This fits the findings that interference is not dependent on the SC structure [10].

Finally, our study highlights the importance of performing meiotic studies in strains of defined and preferentially pure genetic backgrounds. Most significantly, taking into consideration the growing popularity and importance of the conditional gene knock-out approach, it would be highly recommended to use Cre-recombinase mouse lines of the same genetic background as the gene of interest. 

## 4. Experimental Section

### 4.1. Preparation of Slides, Immunofluorescence Staining and Antibodies

We prepared testicular nuclear spreads according to published protocol [46]. We used two mice (6 and 8 weeks old) of each genetic background. Briefly, we combined half a testis of two mice of each strain, minced seminiferous tubules between large forceps in MEM-alpha media (Invitrogen). We transferred the cell suspension to a 10 mL tube and added extra MEM-alpha. We removed cell and tissue debris by a quick spin (400 RPM, 13.5 g) after which we transferred the supernatant to a fresh tube and spun it for additional 7 min at 200 RPM. We removed most of the supernatant with a plastic transfer pipet (Fisher Scientific) and gently resuspended the pellet in the remaining media (~1 mL). We added an equal volume of hypobuffer (17 mM Sodium citrate; 50 mM Sucrose; 30 mM Tris-HCl, pH 8.2) and incubated the cell suspension in the dark for 7 min. After this hypotonic treatment, we spun the suspension at 200 RPM for 7 min. We removed the supernatant with a plastic transfer pipet and gently resuspended the pellet in 2 mL of 100 mM sucrose solution (pH 8.2). We applied a small volume (10–12 µL) of this cell suspension to a paraformaldehyde (PFA)-coated slide (1% PFA, 0.15% triton X-100, pH 9.2). We kept the slides in a humidified chamber for 1.5 h after which we removed the lid and left the slides to dry. We removed dried salts from slides by briefly washing them with 0.08% solution of Photoflow (Kodak) [47].

For antibody staining, we washed slides in solution (PBS-0.05%, Triton X-100, pH 7.4) for 15 min. We subsequently blocked slides in Blocking Buffer solution (PBS-0.05% Triton, 10% normal goat serum, 0.3% BSA) for 20 min in a humidified chamber at room temperature. We diluted the primary antibodies in blocking solution and applied them to the slides. We kept slides at 4 °C overnight in a humid incubation box. We rinsed the slides in the solution (PBS-0.05%, Triton X-100, pH 7.4) and then additionally washed for 15 minutes in PBS, pH 7.4. We then blocked the slides for 30 min with Blocking Buffer without Triton X-100 (PBS, 10% normal goat serum, 0.3% BSA). We then applied secondary antibodies diluted in Blocking Buffer to slides and kept them for 2 hours in a humidified box protected from light. We briefly rinsed slides in PBS, pH 7.4 and then washed them in PBS complemented with DAPI for 15 min protected from light. We rinsed slides in water after DAPI staining and mounted with Vectashield (H-1000).

We used the following primary antibodies in this study: Polyclonal guinea pig SYCP2 (gift of P.J. Wang; used at 1:500), and rabbit polyclonal RPA (gift of C.J. Ingles; used at 1:500). We used Alexa 488 donkey anti-rabbit and Alexa 594 donkey anti-guinea pig (both from Invitrogen; used at 1:1000 dilution) as secondary antibodies.

### 4.2. Imaging and Measurements

We used an Olympus BX61 epifluorescent microscope equipped with an Olympus-ULH100HG camera with a Slidebook 4.1 software package to capture all cells at 1000 × magnification. We only used nuclei with finalized, intact synaptonemal complexes (pachytene spermatocytes). We classified collected images as early pachynema if the width of the SC at the ends was less than 3 times the mid part of the SC. We categorized nuclei with the SC width at the ends 3 times larger than in the middle region of the SC as late pachytene. Thus these nuclei show pronounced deltoid structures at the telomere ends but still have intact SCs. We used MetaMorph (Molecular Devices) to measure SC lengths for each cell. We counted RPA foci manually using MetaMorph features to enhance the contrast between the foci and the SCs. 

### 4.3. RNA Expression Analysis

We used Primer3 program to design PCR primers [48]. We aimed to select primers with a melting temperature close to 60 °C to produce 200 bp amplicons with a similar GC and AT content. We used the following primers: *Sycp1* (TCG CTG ATG ACT GTT CTT GC; AGG TTG AGA AAG CCA AAG CA); *Sycp2* (TTG GGC TCT GGA ATG GAT AG; TGG GAG AAC CAG ACT TCC AC); *Sycp3* (AGG CTG ATC AAC CAA AGG TG; GGG GCC GGA CTG TAT TTA CT); *Smc1β* (AGG CTA CAA CAA TGG CAA CC; ACC ATG AGG GAA AAC GTC AG); *Smc3* (TCC ATA GCA TGC AGA CTT GC; CGC AGG AAC ATT GAA AGG AT); *Rec8* (GCA GCC TCT AAA AGG TGT CG; TGA TAT GGA GGA GGC TGA CC); *Ube2b* (CAT TCC AGC TTT GCT CAA CA; ATC CTG CAG AAT CGA TGG AG); *β-Actin* (TGG GAA TGG GTC AGA AGG ACT; GGG TCA TCT TTT CAC GGT TGG C). We purified total RNA using TRIzol reagent (Invitrogen). We generated cDNA using reverse transcription kit from Invitrogen. We performed quantitative RT-PCR using Quantitect SYBR Green PCR Master Mix (Qiagen) and MJ Opticon Monitor Analysis Software 3.1 on MJ Research Opticon DNA Engine. We repeated the RT‑PCR experiment three times and used the data to generate the graph shown in Figure 6. 

### 4.4. Western Blot Analysis

We prepared testicular protein lysates in Lysis buffer (50 mM Tris-HCl pH 7.5, 150 mM NaCl, 0.1% NP-40, 1 mM DTT, 1 mM PMSF and protease inhibitor cocktail). We used ~30 µg of each total protein lysate for Western blot analysis. We used guinea pig antibodies to REC8 (gift of C. Hoog; used at 1:2000 dilution) and mouse monoclonal antibody to α-Tubulin (clone DM1A, Sigma; used at 1:500 dilution). We performed Western blot image quantification using ImageJ.

## 5. Conclusions

Our results demonstrate that wild-type mice and their genomes may be used to gain additional insights into structure and function of meiotic chromosomes. This approach nicely complements both forward and reverse genetic approaches already applied in this area of reproductive research. At the same time, our findings highlight the importance of performing meiotic studies in mice of pure genetic backgrounds.
